# Reproducibility and validity of a food frequency questionnaire to assess cardiovascular health-related food intake among Mexican adolescents

**DOI:** 10.1017/jns.2022.1

**Published:** 2022-02-02

**Authors:** Ricardo Terminel-Zaragoza, Sonia Vega-López, Gabriela Ulloa-Mercado, Araceli Serna-Gutiérrez, Pablo Gortares-Moroyoqui, Lourdes Díaz-Tenorio, Ana Rentería-Mexía

**Affiliations:** 1 Maestría en Ciencias en Recursos Naturales, Instituto Tecnológico de Sonora, Ciudad Obregón, Sonora, México; 2 College of Health Solutions, Arizona State University, Phoenix, AZ 85004, USA; 3 Departamento de Biotecnología y Ciencias Alimentarias, Instituto Tecnológico de Sonora, Ciudad Obregón, Sonora, México; 4 Departamento de Sociocultural, Instituto Tecnológico de Sonora, Ciudad Obregón, Sonora, México

**Keywords:** AHA dietary targets, Food frequency questionnaire, Mexican adolescents, Reproducibility, Validity, 24 hR, 24 h-recall, AHA, American Heart Association, CVD, cardiovascular disease, FFQ, food frequency questionnaire, F&V, fruits and vegetables, ICC, intra-class correlation coefficient, PCC, Pearson's correlation coefficient, SSB, sugar-sweetened beverage

## Abstract

There is a lack of region-adapted tools to evaluate diet as a risk factor for cardiovascular disease (CVD) in adolescents. The study aim was to evaluate the reproducibility and validity of a paper-based and region-adapted food frequency questionnaire (FFQ) designed to assess CVD-related food and nutrient intakes of adolescents from Northwest México. The study design was cross-sectional. The FFQ was developed in a two-step process: prototype designing and a pilot test, with re-tested in a 3-month period, along with two administrations of 24 h-recall (24 hR). Pearson's and intra-class correlation coefficients (PCC and ICC) were assessed. Bland–Altman plots, limits of agreement and quintile classifications were carried out. Participants (*n* 221) were 53·8 % male, 18·5 ± 0·4 years old. Reproducibility had a median PCC = 0·66 for processed meats, ranging from 0·40 (saturated fat) to 0·74 (fish & shellfish), *P =* 0·001. ICC ranged from 0·53 (saturated fat) to 0·80 (sodium; and nuts, seeds and legumes), *P =* 0·001. Validity comparing FFQ1 *v.* 24 hR mean, PCCs ranged from 0·12 (*P* = 0·06) to 0·95 (*P* = 0·001), and ICC from 0·20 (*P* = 0·048) to 0·88 (*P* = 0·001); comparing FFQ2 *v.* 24 hR mean, PCCs ranged from 0·07 (*P* = 0·25) to 0·46 (*P* = 0·001), and ICC from 0·15 (*P* = 0·106) to 0·58 (*P* = 0·001). The FFQ overestimated the intake of all food groups and nutrients (*P* < 0·05), while Cohen's *κ* showed coefficients lower than 0·20. The proposed FFQ represents a moderately validated tool to estimate CVD-related food and nutrient intakes as a risk factor, which can be used in combination with multiple administrations of 24 hRs, as a critical mean in future interventions intended to reduce cardiometabolic risk in adolescents.

## Introduction

Cardiovascular diseases (CVDs) have been the leading cause of morbidity and mortality in the world over the past decades. A worldwide estimated 17·9 million people died from CVD in 2019, representing 32 % of all global deaths^(1)^. México is not the exception; non-communicable diseases accounted for 80 % of all deaths in México in 2016, with CVD representing 24 % and diabetes 15 % of all deaths^(2)^. In Mexico, the prevalence of medically diagnosed diabetes in adults was 10·3 % (11·4 % in women and 9·1 % in men) in 2018^(3)^.

CVDs have been associated with unhealthy lifestyle patterns^(4,5)^ such as poor diets and physical inactivity, which increase CVD risk through biological, molecular and physiological alterations including inflammation and oxidative stress^(6,7)^. Therefore, the American Heart Association (AHA) recommendations for CVD prevention are mainly focused on reducing lifestyle risk factors^(5)^.

Unlike adults, adolescents are a neglected group in the prevention of CVD, because symptoms normally take 20 years or more to show^(8)^. However, it is well known that atherosclerosis, which is the main underlying cause of CVD, can develop since the first decade of life, especially when a healthy lifestyle and a dietary pattern are not followed^(9,10)^. The prevalence of overweight and obesity in Mexican adolescents increased from 34·9 % in 2012 to 36·3 % in 2016^(11)^. Sonora, a border state in Northwest México, has the highest rates of overweight and obesity in adults (74·7 %) and the second highest in adolescents aged 12–19 years (35·2 %)^(12)^. Despite the increased risk posed by overweight and obesity in adolescents in Sonora, little attention has been paid to assessing lifestyle factors such as physical inactivity, smoking, blood pressure and diet in this group, as well as their relationship with a premature development of CVD^(13)^.

Adolescents tend to undergo changes in their lifestyle during their transition from high school to college/university^(14)^. During this stage, adolescents tend to eat in food establishments close to the school and they are exposed to marketing campaigns for the consumption of unhealthy products. Even if they look for fruits and vegetables (F&V), self-service establishments and internal stores in schools usually do not offer these foods, thus reducing the dietary quality of adolescents^(15)^. This results in the consumption of more processed foods, with more saturated fat and sugar. Furthermore, the time demands from academic and social activities often result in decreases in physical activity levels^(16,17)^. If these habits continue throughout their life, it would be more difficult to adopt healthy habits later in adulthood.

Dietary assessment methods are fundamental for epidemiologic studies and food policy development^(18,19)^, and for understanding eating patterns and their association with disease risk in specific population groups, i.e. adolescents^(20)^. Measuring dietary intake accurately is crucial to understand the role of diet in cardiometabolic diseases, like CVD^(21)^. Currently, one of the most widely used dietary assessment methods is the food frequency questionnaire (FFQ), since it allows obtaining information on long-term habitual consumption, and because it is a relatively cheap, fast and easy-to-apply method^(22)^. The FFQ has surpassed gold standard methods like the 24-h recall (24 hR) since it enables to have greater representativeness of the intake and the seasonal eating patterns^(23,24)^. In this method, the participant indicates the usual frequency of consumption of each of the foods or food groups listed during a given period; the number can be very small, as low as 15 foods, or higher than 250 listed foods^(25)^. Using a pre-existing FFQ designed for a specific demographic group different from that of interest in a given study could lead to inaccurate and unrepresentative results since the characteristics of the questionnaire (i.e. food list and dietary habits) might not be adequate for the target population group^(26)^. Moreover, validated tools for dietary assessment are fundamental to estimate accurate dietary intakes. Therefore, it is essential to have reproducible dietary assessment tools adapted to the target population group and validated with respect to a reference method^(27)^.

One of the best methods to validate FFQ intakes is comparing with nutrient measurements and biochemical indicators since these measurements are assumed to be responsive to intake and independent of self-reports^(28)^. However, biochemical measurements are highly costly and time- and resource-consuming^(19)^, and some biochemical markers are affected by homeostatic regulation and individual biological conditions, which make them inaccurate when correlated to dietary intakes^(29)^. Moreover, these types of studies are typically not used to evaluate FFQs because are not feasible over the time periods to which FFQs typically pertain (e.g., usually a month or a year)^(27)^. Alternatively, FFQs are validated against other self-report dietary assessment instruments, such as 24 hRs. The recalls can be applied altogether with the FFQ, or on different occasions^(30,31)^. Whereas there are multiple FFQs already validated^(32,33)^, they have important limitations because they focus on foods usually eaten in a specific geographic area, and few include foods representative of the diet traits outside of the country of origin. Since it is essential to assess the validity of the selected dietary intake method to determine if it is measuring what people are really eating^(34)^, the aim of the present study is to evaluate the reproducibility and validity of an FFQ designed to assess CVD-related food and nutrient intakes of Northwest Mexican adolescents, according to the AHA-target food groups.

## Materials and methods

### Design and development of the FFQ

We developed a paper-based semi-quantitative FFQ following the methods proposed by Willet *et al.*^(35)^. The design of the FFQ was a two-step process. First, the nutritionist staff developed an FFQ prototype by listing individual foods within each food and nutrient group, including regional foods usually consumed in Northwest México^(36)^. Dietary recommendations for children and adolescents were taken into account^(6)^, and medium portion sizes were adapted according to the Mexican System of Food Equivalents^(37)^. For regional individual foods not included in the Mexican System, the medium portion size was based on their equivalent food groups and/or their weight (either raw or cooked, depending on the usual food intake). Small and large portions were established according to 0·5 and 1·5 medium size, respectively. The FFQ prototype included a list of food groups and nutrients related to CVD based on the following AHA dietary targets: F&V, fish and shellfish (fish & shellfish), sugar-sweetened beverages (SSBs), whole grains, nuts, seeds and legumes, and processed meat for food groups; sodium and saturated fat for nutrients^(5)^. The frequency was set according to the following periods: never, daily, per week (up to 7 d), per month (up to 30 d) or per year (up to 365 d), with a blank space on each option to write the number of times each item was consumed within each selected period. The proposed FFQ covered a year as the longest period of time due that measuring food intake over a period of months to years is more useful when evaluating the association between diet and chronic diseases such as CVD^(26,38)^. In addition, the seasonality of specific food items was accounted for, by adjusting the frequency of intake for the period of the year during which they were consumed, as it is performed in studies with annual questionnaires^(39)^.

On a second step, we pilot-tested the FFQ prototype in sixteen adolescents from 18–19 years of age. Adolescents provided feedback regarding clarity of the instructions and adaptations to the fad oral expressions used by adolescents, types of food included and amounts of some individual foods. Qualitative and quantitative feedback from interviewers and interviewees were used to modify the FFQ prototype. Edits included the modification of instructions and the elimination of individual foods in F&V, fish & shellfish, and nuts, seeds and legumes groups, that participants reported as unknown and not consumed by adolescents. The final FFQ included 152 individual foods and beverages (out of the 159 included in the prototype) that made up the eight food groups (F&V, fish & shellfish, SSBs, whole grains, nuts, seeds and legumes, and processed meat) and two nutrients (sodium and saturated fat).

### Study participants

Participants were enrolled in the reproducibility and validity study if they were freshmen under 20 years of age and if they were willing to complete the FFQs and 24 hRs. Exclusion criteria comprised cardiometabolic diseases, currently taking any medication, pregnancy or breastfeeding, mental disease affecting their ability to participate or previous involvement in the design of this FFQ. Recruitment was done in Náinari Campus from Instituto Tecnológico de Sonora-ITSON (Sonora Institute of Technology) in Cd. Obregón, Sonora, México, over three periods: during the University Induction Program (before classes began), immediately after classes began and during the semester. We found these periods effective for enrolling numerous groups of participants. During each period, we approached students at the university locations explaining the study rationale, aim and procedures. Students were invited to participate, and for those interested, a written study summary was sent to their parents. Written informed consent from parents and written assent from adolescents were obtained, and clinical history and personal data questionnaires were completed. As an incentive, we made an agreement with the university staff to give credits in the ‘Healthy Lifestyle University Program’ to which each student is required to enrol, to those participants who completed the study. Additionally, during the semester, we got support from career directors who allowed us visit classrooms to invite students to the study. Participants completed data collection procedures in person at the Laboratory of Preventive Nutrition and Healthy Eating. For the second administration of the FFQ and 24 hRs, scheduled 3 months after the initial administration, participants were scheduled via cellphone calls or text messages, reminding them the importance of the study for their health, and the credits given as an incentive. The Research Ethics Committee from ITSON approved the study protocol and materials.

### FFQ testing procedure

The FFQ was administered on two occasions (as test–retest) over a 3-month period (August–November 2018) in person, in a pen and paper version. Participants were instructed to indicate the number of times each food was consumed over the previous 12 months and to cross mark their usually consumed portion size: small, medium or large, according to plastic food models (Nasco, Fort Atkinson, WI) and photo-aids (Vitamex Nutrition, Jalisco, México) to help assess portion size.

### 24 hR testing procedure

Data from two 24 hRs were collected. The application of the 24 hRs was from the same day of each application of FFQs; thus, each set of FFQ and 24 hR applications were 3 months apart. On each occasion, the FFQ was administered first, and the 24 hR second. Trained nutritionist staff used established methods for applying the food questionnaires, and the 24 hRs were administered following the five-step automated multiple-pass method developed by the USDA^(40–42)^. The 24 hR included three final questions asking if it was a typical food-consumption day, the adherence to a special diet and supplement intake. For the administration of questionnaires, staff used the previously mentioned food models and pictures for helping participants identify foods and portions.

### FFQ and 24 hR data management

For calculating food group and nutrient amounts, data from the FFQs and 24 hRs were captured in a web app using the Mexican System of Food Equivalents^(37)^, and those not found were checked in the USDA Global Branded Food Products Database^(43)^.

After the FFQs were checked for completeness or missing values, data were double-checked and captured in a Microsoft Excel spreadsheet (Microsoft Co., Redmond, WA, USA) with established equations using portion size and frequency consumption to calculate either daily or weekly intake from individual foods, and then summed for each food group. For F&V (cups), sodium (mg), whole grains (oz) and saturated fat (g and %), data were calculated by day. Fish & shellfish (oz), SSBs (oz), nuts and seeds (oz), legumes (cups) and processed meat (g) were calculated by week. Outliers, defined by ±3 sd, were trimmed from each group.

Food items from each 24 hR were recorded into separated Microsoft spreadsheets with the same calculations as the FFQ, so food items were matched to the food group intakes defined in the FFQ. For example, ‘Chocolate milk-banana-oatmeal shake’ was assigned to banana in F&V, oatmeal in whole grains and chocolate milk in SSBs, according to the AHA dietary target food groups and nutrients of interest for preventing CVD^(5)^. The portion size from the 24 hRs and frequencies (in the first or second application of the 24 hRs) were used to calculate either daily or weekly consumption. Each final intake from food groups from the 24 hRs was calculated in the same food groups and units as the FFQ.

### Statistical analysis

Raw data for food groups were checked for normal distribution according to skewness and kurtosis tests, and the Kolmogorov–Smirnov test^(44)^. Statistical and graphical assessment methods were used to determine the most appropriate transformation to achieve normal distribution. Squared root transformations and/or log transformations were used when appropriate. Paired-samples *t*-tests were used for comparing the food intake from FFQ1 and FFQ2, and for FFQ mean and 24 hR mean, as well as for the 24 hR mean against FFQ1 and FFQ2, separately.

Reproducibility (test–retest reliability) was assessed by comparing the food group, sodium and saturated fat intakes from FFQ1 and FFQ2. Validity was performed comparing the mean of the 24 hRs against FFQ1 and FFQ2, separately. For both reproducibility and validity, Pearson's correlation coefficients (PCCs) (95 % CI) were calculated after statistical adjustment for between-person variation. For within-person reliability, intra-class correlation coefficients (ICCs) (95 % CI) were calculated to consider variations caused by day-to-day within-person variations^(31,45)^ and using the ratio of the within- and between-person variances and *n* as the number of replicates per person for the given intake. Within-person and between-person variances were calculated from the replicated 24 hRs. ICC was used for validity as well since it is a useful parameter to compare two different measurement methods^(46,47)^. Bland and Altman plots were used to graphically examine the agreement for food groups and nutrient intakes between the FFQs and 24 hRs and to estimate the 95 % limit of agreement (LOA), in which 95 % of all differences between methods are expected to fall. Differences in absolute intakes (FFQ − 24 hR) and mean food group and nutrient intakes from both dietary instruments were calculated (agreement at group level). Mean agreement for each food group and nutrient was estimated as the mean of all individual differences between the FFQs and 24 hRs (∑FFQ – 24 hR)/*n*, while LOA was calculated as mean difference ± (sd*1·96). All food groups and nutrient intakes were transformed to their natural logarithms before analyses due to the usual skewness in intake distributions. We also examined whether the agreement was constant across the range of intake by estimating the regression slope of differences (*ß*) between the FFQ and the 24 hR, that is, regressing the average of the two methods on their differences^(48)^. The distribution of food groups and nutrient intakes was also categorised into quintiles to evaluate agreement at the individual level. The proportion of participants classified by the FFQ mean that were into the same, adjacent or extreme quintile of the 24 hR mean was used to calculate the degree of misclassification, and Cohen's *κ* was calculated and classified according to the criteria by Altman^(49)^. All data were analysed using the Statistical Package for Social Sciences, Version 21·0 (SPSS Inc., Chicago, IL, USA), and results were considered statistically significant with a *P* value of <0·05.

## Results

From 264 recruited adolescents, 84·7 % completed both administrations of the FFQs and 24 hRs, and were included in the present study (*n* 221). Participants were 53·8 % male and 46·2 % female, 18·5 ± 0·4 and 18·4 ± 0·4 years of age for male and female, respectively, at the time of screening. Table 1 shows the intakes for CVD-related food groups and nutrients from both FFQs separately and the mean from the 24 hRs. Intakes reported in the FFQ2 were slightly higher than the FFQ1 in eight out of ten categories, except for SSBs [oz/week (wk)] and saturated fat (kcal/d and %/d), both of which were lower (*P* < 0·05). All the FFQ mean food group and nutrient intakes were higher than the 24 hR mean intakes (all *P* < 0·001), consistent with reported differences between these two methods in the adolescent population ^(16,42,50,51)^. It is noticeable that mean intakes reported through the FFQ, although overestimated, did not meet most of AHA dietary targets (eight out of ten), except for fish & shellfish (oz/wk), nuts (oz/wk) and legumes (cups/wk) groups. For harmful food groups like sodium, SSBs, processed meats and saturated fat, FFQ intakes were higher, and 24 hR intakes were lower than the AHA target (Table 1).

**Table 1.** Intakes of CVD-related food groups and nutrients from FFQ1, FFQ2, mean of FFQs and 24 hRs in Northwest Mexican adolescents (*n* 221)

CVD-related food groups and nutrients	AHA target	FFQ1		FFQ2		*P* value^a^	FFQ mean		24 hR mean		*P* value^b^
		Mean	sd	Mean	sd		Mean	sd	Mean	sd	
Fruits & vegetables (cups/d)	≥4·5 cups/d	3·2	1·7	4·0	3·1	<0·001	3·6	2·2	0·17	0·15	<0·001
Fish & shellfish (oz/wk)	≥7 oz/wk	17·1	12·9	18·7	22·6	0·285	18·0	14·9	0·8	1·8	<0·001
Sodium (mg/d)	≤1500 mg/d	2162·0	1498·8	2334·6	2536·6	0·276	2248·3	1720·1	1191·7	116	<0·001
SSBs (oz/wk)	≤36 fl oz/wk	132·3	90·7	107·9	64·5	<0·001	120·1	68·2	18·3	12·1	<0·001
Whole grains (g/d)	3 or more 1-oz-eq servings/d	2·5	1·6	2·6	1·7	0·583	2·5	1·5	0·5	0·3	<0·001
Nuts & seeds (oz/wk)	1 oz	1·1	1·3	1·4	2·0	0·003	1·3	2·5	0·02	0·08	<0·001
Legumes (cups/wk)	1/2 cup	1·4	1·9	1·5	2·1	0·045	1·4	2·0	0·3	0·3	<0·001
Nuts, seeds & legumes mean (servings/wk)	≥4 servings/wk (nuts/seeds: 1 oz; legumes 1/2 cup)	2·5	2·5	3·1	3·7	0·001	2·2	2·8	0·3	0·3	<0·001
Processed meats (oz/wk)	≤3·5 oz/wk	20·4	13·2	21·0	15·7	0·051	20·7	18·6	11·91	2·3	<0·001
Saturated fat (kcal/d)		175·8	87·7	139·2	54·6	<0·001	157·5	61·8	24·1	8·2	<0·001
Saturated fat (%/d)	≤7 % energy/d	10·5	6·1	9·4	4·9	0·015	10·0	4·6	7·5	0·5	<0·001

CVD, cardiovascular disease; FFQ, food frequency questionnaire; 24 hR, 24-h recall; AHA, American Heart Association; wk, week; SSB, sugar-sweetened beverage.

a Paired-samples *t*-test between FFQ1 and FFQ2.

b Paired-samples *t*-test between FFQ mean and 24 hR mean.

### Reproducibility study

Results from the reproducibility study of the FFQs are shown in Table 2. The PCCs between the two FFQs ranged from 0·40 to 0·74 for the total sample, with 0·40 to 0·80 for men and 0·39 to 0·77 for women (all *P* < 0·001). The median PCC between the two applications of the FFQ was *r* = 0·66 (*P* < 0·001) for processed meats. Saturated fat (%/d) was the dietary target with the lowest PCC for the total sample (*r* = 0·40; *P* < 0·001). Concerning ICC, all values were greater than 0·50, ranging from 0·53 for saturated fat (%kcal/d) to 0·80 for sodium (mg/d), *P* = 0·001. The food groups with high ICC values also showed high PCC values.

**Table 2.** Reproducibility of the FFQ for CVD-related food groups and nutrients using PCCs and ICCs in Northwest Mexican adolescents (*n* 221)

CVD-related food groups and nutrients	PCC men (*n* 119)	PCC women (*n* 102)	PCC total (*n* 221)	ICC total (*n* 221)
Fruits & vegetables (cups/d)^a^	0·68*	0·77*	0·71*	0·79*
Fish & shellfish (oz/wk)^a^	0·78*	0·70*	0·74*	0·77*
Sodium (mg/d)^a^	0·76*	0·62*	0·70*	0·80*
SSBs (oz/wk)	0·52*	0·51*	0·55*	0·65*
Whole grains (g/d)	0·63*	0·62*	0·63*	0·77*
Nuts & seeds (oz/wk)^a^	0·80*	0·59*	0·71*	0·77*
Legumes (cups/wk)^a^	0·57*	0·62*	0·59*	0·65*
Nuts, seeds & legumes mean (servings/wk)^a^	0·73*	0·65*	0·70*	0·80*
Processed meats (oz/wk)^a^	0·67*	0·62*	0·66*	0·76*
Saturated fat (kcal/d)^a^	0·51*	0·50*	0·50*	0·55*
Saturated fat (%kcal/d)^a^	0·40*	0·39*	0·40*	0·53*

FFQ, food frequency questionnaire; CVD, cardiovascular disease; PCC, Pearson's correlation coefficient; ICC, intraclass correlation coefficient; wk, week; SSB, sugar-sweetened beverage.

a Squared root-transformed value.

* *P* < 0·001.

### Validation study

Table 3 shows the validity between the 24 hR mean intake and the FFQ1 and FFQ2. The median PCC for FFQ1 was saturated fat (kcal/d) *r* = 0·19 (*P* = 0·004); and for FFQ2 was saturated fat (kcal/d) and whole grains, both with *r* = 0·20 (*P* = 0·002). The highest PCCs for both FFQs were for saturated fat (%kcal/d), with *r* = 0·95 (*P* = 0·001) for FFQ1, and *r* = 0·46 (*P* = 0·001) for FFQ2. The lowest PCCs for both FFQs were for sodium, with *r* = 0·12 (*P* = 0·060) for FFQ1, and *r* = 0·07 (*P* = 0·250) for FFQ2. Despite the low PCC between the FFQs and the 24 hR mean, most coefficients were statistically significant, except for sodium in both FFQs; and legumes (*r* = 0·10, *P* = 0·138) and nuts, seeds and legumes group for FFQ2 (*r* = 0·09; *P* = 0·171). Concerning ICC between each FFQ and 24 hR means, ICC ranged from 0·20 to 0·88 (*P =* 0·048 and 0·001, respectively) for FFQ1, and from 0·15 to 0·58 (*P =* 0·106 and 0·001, respectively) for FFQ2. The highest ICCs for both FFQs were saturated fat (%kcal/d), with *r* = 0·88 (*P* = 0·001) for FFQ1, and *r* = 0·58 (*P* = 0·001) for FFQ2. The lowest ICCs were fish & shellfish for FFQ1 with *r* = 0·20 (*P =* 0·048), and sodium for FFQ2 with *r* = 0·15 (*P* = 106).

**Table 3.** Validity between the FFQ for CVD-related food groups and nutrients and mean intake of 24 hRs using PCC in Northwest Mexican adolescents (*n* 221)

CVD-related food groups and nutrients	FFQ1 *v.* 24 hRs				FFQ2 *v.* 24 hRs			
	PCC	*P* value	ICC	*P* value	PCC	*P* value	ICC	*P* value
Fruits & vegetables (cups/d)^a^	0·29	0·001	0·33	0·002	0·34	0·001	0·31	0·003
Fish & shellfish (oz/wk)^a^	0·14	0·036	0·20	0·048	0·15	0·024	0·21	0·035
Sodium (mg/d)^a^	0·12	0·060	0·22	0·030	0·07	0·250	0·15	0·106
SSBs (oz/wk)	0·17	0·010	0·21	0·036	0·23	0·001	0·33	0·001
Whole grains (g/d)	0·24	0·001	0·42	0·000	0·20	0·002	0·35	0·001
Nuts & seeds (oz/wk)	0·22	0·001	0·32	0·002	0·25	0·001	0·33	0·001
Legumes (cups/wk)^a^	0·17	0·011	0·27	0·008	0·10	0·138	0·21	0·035
Nuts, seeds & legumes (servings/wk)^a^	0·18	0·007	0·31	0·003	0·09	0·171	0·21	0·041
Processed meats (oz/wk)^a^	0·22	0·001	0·32	0·002	0·30	0·001	0·30	0·003
Saturated fat (kcal/d)^a^	0·19	0·004	0·29	0·006	0·20	0·002	0·22	0·032
Saturated fat (%kcal/d)^a^	0·95	0·001	0·88	0·001	0·46	0·001	0·58	0·001

FFQ, food frequency questionnaire; CVD, cardiovascular disease; 24 hR, 24-h recall; PCC, Pearson correlation coefficient; ICC, intraclass correlation coefficient; wk, week; SSB, sugar-sweetened beverage.

a Squared root-transformed value.

**Fig. 1.**
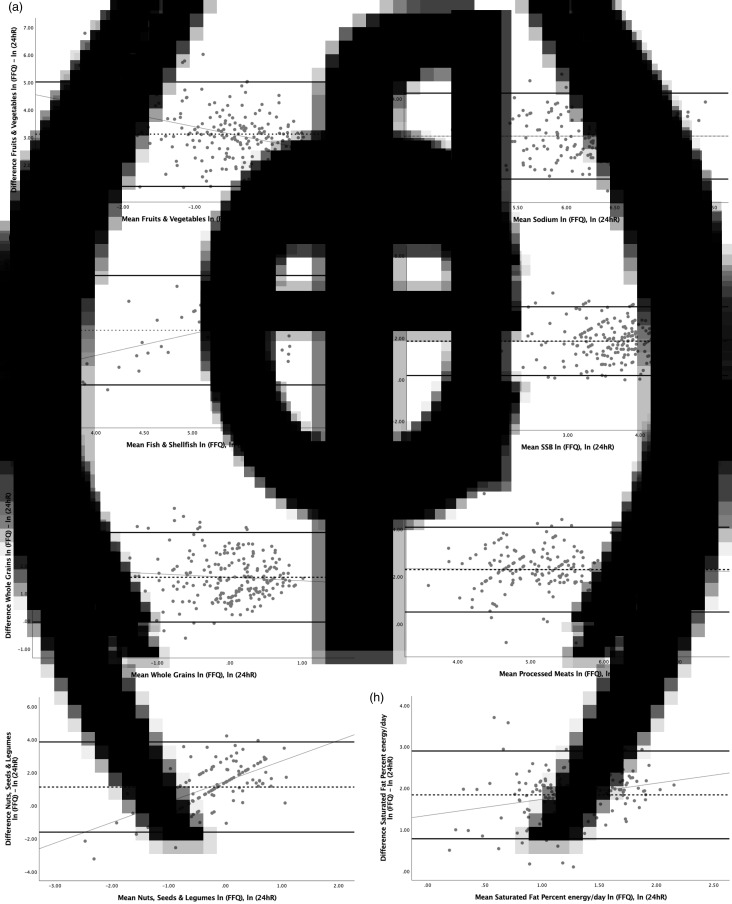
Bland–Altman plots and 95 % limits of agreement (LOA) for CVD-related food groups and nutrients. (a) Fruits & vegetables, (b) fish & shellfish, (c) sodium, (d) SSB, (e) whole grains, (f) nuts seeds & legumes, (g) processed meats and (h) saturated fat percent energy per day. Mean of differences between ln(FFQ) – ln(24 hR) (dotted line); 95 % LOA (thick solid line); regression slope with *P* > 0·05 for sodium, SSB, whole grains and processed meats (thin solid line). SSB, sugar-sweetened beverage; ln, natural logarithm; FFQ, food frequency questionnaire; 24 hR, 24-h recall.

All food groups and nutrients showed average agreement between FFQs and 24 hRs greater than 100 % (Table 4). The FFQ overestimated the intake of the total food groups and nutrients (*P* < 0·05). For fish & shellfish, nuts, seeds and legumes, and saturated fat, agreement between the FFQ and the 24 hR was significantly poorer at high levels of intake (significant positive slope in differences in Fig. 1), and for F&V agreement was significantly poorer at low intakes (significant negative slope in differences in Fig. 1). For sodium, SSBs, whole grains and processed meats, agreement was non-significant with *β* coefficients close to 0, which means that the variance of the differences does not vary across the range of means (*P* > 0·05). Widest ranges of LOA were observed for F&V, processed meats and saturated fat, and less wide for SSBs, whole grains, and nuts, seeds and legumes. Fig. 1 presents the Bland–Altman plots with the mean agreement, 95 % LOA and regression slopes of differences, in which the slopes graphically indicated no proportional bias for sodium, SSBs, whole grains and processed meats. This means that although the FFQ overestimated their intakes, the variances of the differences were constant across the range of means (*P* > 0·05).

**Table 4.** Mean% agreement and 95 % LOA between the FFQs and 24 hRs for CVD-related food groups and nutrients in Northwest Mexican adolescents (*n* 221)

CVD-related food groups and nutrients	Mean% agreement^b^	(95 % CI)	LOA^c^ (%)	Slope^d^	
				(*ß*)	*P* value^e^
Fruits & vegetables (cups/d)	226·9	(198·7, 258·8)	34·2−1505	−0·522	0·0001
Fish & shellfish (oz/wk)	156·9	(141·7, 177·6)	17·7−575·1	0·919	0·0001
Sodium (mg/d)	112·5	(99·5, 127·3)	18·3−692·3	0·002	**0**·**989**
SSBs (oz/wk)	164·1	(157·1, 171·8)	12·2−337	0·011	**0**·**925**
Whole grains (g/d)	148·7	(143·6, 154·6)	10·4−247·3	−0·126	**0**·**22**
Nuts, seeds & legumes mean (servings/wk)^a^	130·4	(123·8, 138·7)	50·5−466·2	1·244	0·0001
Processed meats (oz/wk)	198·9	(186·1, 1113·8)	16·5−1595·6	−0·026	**0**·**828**
Saturated fat (%kcal/d)	162·7	(158·4, 167·5)	121·8−1183	0·388	0·0001

LOA, limit of agreement; FFQ, food frequency questionnaire; 24 hR, 24-h recall; CVD, cardiovascular disease; CI, confidence intervals; wk, week; SSB, sugar-sweetened beverage.

a Nuts, seeds & legumes were not individually analysed since differences in intakes for the majority of individuals were ≤0 and their natural logarithm do not exist; thus, many observations were considered as missing values.

b Exp(mean(FFQ − 24 hR)), all nutrient data were natural log-transformed.

c 95 % LOA [mean difference ± *t*_(*n*−1, 0·05)_ (sd differences)].

d Slope of the average of methods regressed on the difference between the methods (H_0_: *β* = 0, *α* = 0·05).

e Bold *P* values represent slopes (*ß*) that are close to 0 and are non-significant, which means that the variance of the differences does not vary across the range of means; thus, there is no proportional bias.

Table 5 shows the ranking analysis in which 19–30 % of participants were classified by the FFQ mean into the same quintile, approximately 30–40 % into adjacent, and 2 to almost 20 % into opposite quintiles of the CVD-related food groups and nutrient intakes. The level of agreement between the two methods was poor for all food groups and nutrients, as Cohen's *κ* coefficients were lower than 0·20, according to the Altman^(49)^.

**Table 5.** Percentage of subjects classified by the FFQ mean into the same or different quintile of consumption and Cohen's *κ* coefficient as measured by the 24 hR mean for CVD-related food groups and nutrients in Northwest Mexican adolescents (*n* 221)

CVD-related food groups and nutrients	Same quintile (%)	Adjacent quintile (%)	Opposite quintile (%)	Cohen's *κ*	
				Coefficient	*P* value^b^
Fruits & vegetables (cups/d)	29·4	41·2	5·0	0·102	**0·003**
Fish & shellfish (oz/wk)	20·4	32·6	19·5	0·025	0·312
Sodium (mg/d)	19·0	36·2	4·5	−0·014	0·665
SSBs (oz/wk)	29·0	29·9	4·1	0·107	**0·002**
Whole grains (g/d)	24·0	32·1	2·3	0·044	0·192
Nuts, seeds & legumes mean (servings/wk)^a^	19·9	34·4	7·2	0·029	0·323
Processed meats (oz/wk)	26·2	33·5	5·0	0·071	**0·034**
Saturated fat (%kcal/d)	28·5	35·3	4·5	0·104	**0·002**

FFQ, food frequency questionnaire; 24 hR, 24-h recall; CVD, cardiovascular disease; wk, week; SSB, sugar-sweetened beverage.

a Nuts, seeds, & legumes were not individually analysed since differences in intakes for the majority of individuals were ≤0.

b Bold *P* values represent that the *κ* value is significantly different from 0. This does not imply statistically significant agreement.

## Discussion

We developed a semi-quantitative FFQ proposed to estimate CVD-related food intake and tested its reproducibility and validity. For reproducibility, intakes of food groups from two separate FFQs applied within a 3-month period were correlated. Validity was assessed comparing each FFQ with the mean of two 24 hRs. Regarding the 159 food items initially considered within ten food groups and nutrients, 152 food items were finally included, with seven items excluded in the FFQ and data analysis for not having representativeness on the average of the respective food-group intake, as they were food items reported as unknown by adolescents.

Intakes from the FFQ values were higher than the 24 hR values, and PCCs were low for most of the food groups and nutrients when comparing the 24 hR with the FFQ, which is consistent with other studies^(16,39,42)^. Data from short-term dietary instruments such as 24 hR have shown great variability from day to day, resulting in low intakes of certain foods eaten sporadically, especially when few repetitions of 24 hR are used^(16)^. Besides, people tend to overestimate food intakes when presented with a large list of items within the questionnaire, such as the FFQ. Moreover, asking participants to retrospectively report their intakes over long periods of time (e.g. 1-year FFQ) is a complex task that may contribute to people tending to overestimate and others to underestimate their intakes, especially for foods that are episodically consumed^(27)^. Therefore, large differences in reported intakes may be responsible for such large standard deviations between the results of the FFQ mean. Other error sources could be the difficulty in conceptualising the assigned portion sizes, despite the use of food models and photo-aids to help participants visualise portion sizes^(16,27)^.

Results from the present study demonstrated that the FFQ obtained a high reproducibility for the majority of the food groups and nutrients, with the exception of saturated fat (%/d). The median PCC of 0·66 (range 0·40–0·74) from the present study is comparable in magnitude to those reported in previous studies in adolescents, with PCC = 0·34 and PCC = 0·85 reported by Neuhouser *et al.*^(52)^ and by Watson *et al.*^(20)^, respectively, and also for Mexican adult population with PCC = 0·27 and PCC = 0·77 by Hernández-Avila *et al.*^(53)^ and by Macedo-Ojeda *et al.*^(23)^, respectively. The median ICC in the present study was 0·65 (range 0·53–0·80), which is considered acceptable for an FFQ (0·50–0·70)^(38,54)^.

For validity, a small variation was seen in the PCCs between each FFQ and the 24 hR. Among the food groups and nutrients in both FFQs, all had PCCs ranged from 0·07 to 0·34, exerting a poor to fair validity, except for saturated fat (%/d) which ranged excellent to moderate validity (0·95 for FFQ1 and 0·46 for FFQ2). When analysing ICCs, FFQ1 showed a fair to excellent validity (0·20–0·88), and poor to moderate for FFQ2 (0·15–0·58). ICC had a modestly higher correlation than PCC. According to their PCC during validation, fish & shellfish (FFQ1, *r* = 0·14), sodium (FFQ1, *r* = 0·12; FFQ2, *r* = 0·07), legumes (FFQ2, *r* = 0·10), and nuts, seeds and legumes (FFQ2, *r* = 0·09) were the food groups with the poorest validity. These food groups are used in the preparation of Northwest Mexico traditional dishes of importance due to cultural heritage^(36,55)^. However, it is possible that the consumption of these foods did not occur on the days of the 24 hR. This sort of limitation is present when validating an FFQ with a reference method that does not cover the same time interval, like the 24 hR^(56)^.

Although some studies have reported the validation of FFQs for the Mexican population, these questionnaires do not include foods typically consumed in the Northwest region of México. One of these studies by Hernandez *et al.* validated a 116-item semi-quantitative FFQ against 16–24 hRs, but only included women residing in Mexico City^(53)^. Reproducibility by ICC ranged from 0·43 to 0·60, similar to our validation results in Table 2. The first application of the FFQ showed a validity range of 0·13–0·52, and the second FFQ was slightly higher (0·19–0·56). Moreover, to our knowledge, there are no previously validated FFQs, especially designed for estimating Mexican adolescent food intake from this region.

In a study by Imaeda *et al.*^(57)^, authors evaluated the reproducibility and validity of a short FFQ for twenty food-group intakes in the middle-aged Japanese population, compared with a 3-d dietary record. For reproducibility, Spearman's rank correlation coefficients were 0·61 (range 0·38–0·86) for men and 0·66 (0·45–0·84) for women, while for validity, Spearman's rank correlation coefficients were 0·51 (0·17–0·76) for men and 0·47 (0·23–0·77) for women. The FFQ proposed by these authors demonstrated higher reproducibility and validity than those found in the present study. Among the main differences between the study by these authors and our present study is the reference method, in which a 3-d dietary record is a more reliable method that depends less on the memory of the interviewees^(57)^. Similarly, when compared with other studies for the evaluation of food groups, results from our research were similar in reproducibility than an FFQ designed for New Zealand adolescents (PCC of 0·26–0·92)^(58)^, and higher than children and adolescents nationally representative from Germany (0·01–0·30)^(59)^ and Australia (0·34–0·51)^(20)^. The FFQ developed in the present study had lower PCC and ICC than results from a 19-item FFQ assessing snacks by food groups in American adolescents, which reported reproducibility ranged from 0·72 to 0·85, and validity from 0·56 to 0·87^(52)^. One of their strengths was the short length of the questionnaire since they assessed only a specific food group, but their sample size (*n* 42) was relatively low compared with our sample size (*n* 221). Moreover, our validation results were similar in range to those studies who also validated an FFQ by food groups in adolescents (0·04–0·72)^(58)^, children and adolescents from Germany (0·22–0·69)^(59)^, and Australia (0·13–0·37)^(20)^; German adolescents and adults (0·15–0·80)^(60)^, and Switzerland adults (0·28–0·71)^(61)^.

Validation against 24 hR is one of the most used tools when validating FFQs for both adolescents and adults^(59,60,62–64)^. However, 24 hR as well as other dietary methods are not free from random errors that may interfere with the precision and accuracy of the measurement protocol^(30)^. Encompassing a wide range of food items from different food groups, this FFQ may offer a viable approach to assess AHA target dietary groups in large studies of adolescents. Our proposed FFQ was not merely adapted from other studies but was designed to include typical foods from the region, and we consider that the final food item list sufficiently covers the common regional foods consumed by Northwest Mexican adolescents, which are food items of cardiovascular interest and it is hence suitable to assess food group intakes in this age group.

Some of the strengths in the present study are the administration of the questionnaires directly to the adolescents (and not to their parents) as well as the 3-month interval re-administration which may have led to the good reproducibility results^(20)^. Its design was region-adapted for Northwest Mexican adolescents, which allowed us to evaluate regional and seasonal foods in the FFQ for the whole year seasonality with the application of the FFQ in each season (there is only summer and winter in our region). The sample had an equitable distribution of male and female participants, and the sample size was relatively large compared with other studies validating FFQs for adolescents^(52,58,63)^.

Similar to other studies assessing the reproducibility and validity of FFQs for children and adolescents, the present study has limitations worth discussing. An important limitation is associated with the amount of time that each instrument assesses, as this did not allow for both methods to capture the same construct over the same period of time^(27,65)^. Short-term dietary tools show wide day-to-day variability, thus increasing the within-person variation. Therefore, in the case of the 24 hR used as the reference method, a recommendation is to have a large number of administrations so that the usual intake can be estimated^(27,65)^. Carroll *et al.*^(66)^ found that four to six administrations of the 24 hR is optimal for most nutrients and food groups, and that the combined use of multiple 24 hRs and FFQ data sometimes provides data superior to the use of either method alone, especially for foods that are not regularly consumed. However, the present study only included two 24 hRs, administered on the same days as the two FFQ administrations. More 24 hR were not administered for multiple reasons: (1) to avoid the challenges inherent to collecting data from adolescents, who may be reluctant to share information about their behaviours; (2) to prevent participant burden, mental fatigue and boredom, all of which could influence participant responses; and (3) to dedicate resources towards maximising the study sample size^(67,68)^. It is possible that only having two 24 hRs together with having an extensive number of individual foods in the FFQ may have synergistically affected our results, with the FFQ overestimating and the 24 hR underestimating habitual food intake^(27,42,69,70)^.

Another important limitation is associated with the large standard deviations between the results of the FFQs, reflecting between-person variations. As previously mentioned, many of the foods listed in the FFQ were consumed episodically, resulting in a large margin of intake by the majority of the participants due to the high day-to-day variation of foods such as fish and whole grains^(56)^. Other factors that could have influenced the between-person variations could be intake-related biases (i.e. the quantity of intake is related to discrepancies in reporting^(27,71)^ and the high number of individual items within each food group and nutrient). Following these recommendations, our proposed FFQ could be used to measure usual intake for CVD-related foods, especially for sodium, SSBs, whole grains and processed meats, as the food groups and nutrients that showed no proportional bias using Bland–Altman plots and linear regression, as long as proper attention is paid to the best practices of conducting dietary interviews^(27)^.

Another limitation is that the present study only included college students, who may be different from other groups of adolescents, limiting the external validity of the study. The ‘freshmen transition’ is a transformational process experienced by individuals during the critical period of university entry. Successful adaptation for university freshmen involves positive developments in diverse aspects, such as academic abilities, establishing and maintaining relationships and an individual life philosophy, cultivating self-identity, maintaining physical and mental health, among others, all of which could potentially influence survey responses^(72)^. Moreover, self-reported dietary assessment is still a subjective measure and prone to errors since it relies on short- and long-term memory^(30)^. Despite these limitations, the proposed FFQ represents a well-reproducible and moderately validated questionnaire to assess food frequency intakes for specific CVD-related food groups and nutrients in adolescents from this region of México.

## Conclusions

The cultural and gastronomic differences from Northwest Mexico, compared with the rest of the country, make it difficult to have national generic tools for the dietary evaluation of specific population groups. The proposed FFQ, which was tailored to consider adolescent food habits specific to the Northwestern region of Mexico, represents a moderate-validated, well-adapted and cost-effective tool to assess diet as a risk factor for CVD among adolescents, which could be used in combination with multiple administrations of 24 hRs in case that more accurate data are needed. Finally, the designed FFQ can be used as critical means to estimate consistent changes over time in food intake during future interventions intended to reduce cardiometabolic risk in this young group. The designed FFQ can help Northwest Mexican nutrition researchers establish regional nutrition policies that consider regional food lifestyle, in usual environments of adolescents such as schools, colleges and universities.
